# Acupuncture for acute moderate thalamic hemorrhage: randomized controlled trial study protocol

**DOI:** 10.1186/s12906-017-1614-6

**Published:** 2017-02-15

**Authors:** Chengwei Wang, Chao You, Lu Ma, Mengyue Liu, Meng Tian, Ning Li

**Affiliations:** 1 Department of Integrated Traditional and Western Medicine, West China Hospital, Sichuan University, Chengdu, China; 2 Neurosurgery, West China Hospital, Sichuan University, Chengdu, China

**Keywords:** Acupuncture, Intracranial hemorrhage, Moderate thalamic hemorrhage, RCT

## Abstract

**Background:**

Thalamic hemorrhage (TH) is a neurological insult with a high rate of morbidity and mortality. Moderate TH (10–30 ml) accounts for more than half of all TH. Treatment remains controversial. The role of acupuncture in patients with moderate TH is not clear.

**Methods:**

We will conduct a single-center, randomized, parallel group, and assessor-blinded clinical trial. A total of 488 patients with moderate TH will be randomly assigned to one of eight groups: 10–15 cc left sided TH study group (*N* = 61) and a corresponding control group (*N* = 61), 10–15 cc right sided TH study group (*N* = 61) and a corresponding control group, 15–30 cc left sided TH study group (*N* = 61) and a corresponding control group (*N* = 61), and 15–30 cc right sided TH study group (*N* = 61) and a corresponding control group. Study groups will receive acupuncture in addition to standard treatment, while control groups will receive standard treatment alone. The primary outcome will be change in National Institutes of Health Stroke Scale scores at 30 and 90 days after TH. The secondary outcomes will be death or major disability, defined as a score of 3 to 6 on the modified Rankin scale (in which a score of 0 indicates no symptoms, a score of 5 indicates severe disability, and a score of 6 indicates death) at 90-days, need for surgery at 30-days, Glasgow Outcome Scale (GOS) score at 90-days following TH onset, and the results of several additional group specific tests. The rate of adverse events will then be compared between the groups.

**Discussion:**

This study will attempt to answer the question of whether or not acupuncture can improve neurologic outcome following moderate TH.

**Trial registration:**

Chinese clinical trial registry (ChiCTR-IOR-16008362)

## Background

Spontaneous intracerebral hemorrhage (ICH), non-iatrogenic ICH without trauma, is the second most common and the most devastating form of all strokes with the poorest prognosis [1, 2]. Approximately 15% of all cases of spontaneous ICH occur in the thalamus [3–5]. The thalamus is a deep brain structure and is an important center for the transmission of neural signals [3, 6]. Due to its anatomical location and the vital functions that it performs, the decision of conservative or surgical management for TH remains controversial [2, 7, 8]. Management is influenced mainly by the clinical experience of the treating surgeon and the volume of the hematoma [9]. Patients with minor TH, especially volumes less than 10 cc, usually obtain favorable outcomes when treated with conservative measures. However, patients with major TH, greater than 30 cc, have mortality rates of more than 80%, no matter what treatment is selected [10]. Moderate TH (10–30 mL) constitutes more than half of all cases of TH, and these patients have a mortality rate of more than 30% [11]. As moderate TH is the most common form of TH, its prognosis carries the greatest weight in calculating the prognosis of all forms of TH. In patients with moderate TH, conservative treatment alone versus surgical treatment is naturally a difficult decision to make. Because surgical treatment is invasive, adjacent structures may be damaged and neural pathways may be disrupted during surgical evacuation of a hematoma, thereby aggravating the degree of neurologic deficits or creating new ones. Meanwhile, conservative treatment alone does not offer the involved thalamic tissue the same type of relief that evacuation offers and is therefore felt to be a limited strategy in improving neurologic function. The limitations of both therapeutic strategies provide considerable room for better approaches or combinations of approaches.

Two recognized mechanisms of brain injury from ICH have been established [12, 13]. Primary injury happens at the time of initial hemorrhage when direct damage to brain tissue occurs either secondary to the hematoma itself or to its mass effect on surrounding brain tissue. Secondary injury happens at some time following initial hemorrhage and includes neuronal death from either the inflammatory response to the hematoma, the release of toxic materials and other cytokines, the breakdown of the brain blood barrier, apoptosis, or the development of edema. While the pathophysiology of ICH provides many potential approaches for its treatment, enlargement of the hematoma and the development of surrounding edema have been identified as major contributors to the high morbidity and mortality of ICH and have thus been the major focus of potential treatment strategies [14–17].

Acupuncture, one of the major branches of Traditional Chinese Medicine (TCM), has long been used to treat acute stroke, including hemorrhagic stroke, in China as well as in many other East Asian countries. Acupuncture’s utility in the treatment of non-hemorrhagic stroke, which is now documented by the WHO (World Health Organization, 2002), has been widely researched [18]. In recent years, a number of studies using modern technologies have shown that acupuncture may have neuroprotective effects following hemorrhagic stroke as well [19–22]. One preclinical systematic review of 19 animal studies showed that GV20 (Baihui) based acupuncture can improve neurologic outcome by regulating the inflammatory response to a hematoma, inhibiting neuronal apoptosis, reducing cerebral edema, maintaining ATP supply, promoting nerve regeneration, maintaining neuronal membrane integrity, and promoting hematoma resorption [23]. In another animal study, acupuncture at the DU20 acupoint was reported to have a neuroprotective effect on cerebral hemorrhage by inhibiting Notch-Hes signaling in rat basal ganglia [24]. It has also been reported that electro-acupuncture treatment at the Zusanli (ST36) acupoint may accelerate ICH-induced angiogenesis via the up-regulation of the HIF-1α protein and therefore may enhance recovery following hemorrhagic cerebral injury [25]. A separate study confirmed that treatment at the ST36 acupoint may aid recovery following central nervous system intracerebral hemorrhage in rats [26]. Although animal studies have led to progress in determining how acupuncture exerts its benefits in the treatment of acute hemorrhagic stroke, the determination of its clinical value remains incompletely assessed [27–30].

To our knowledge, no organized study has reported on the effects of acupuncture in patients with acute moderate TH. Specifically, no prior study has examined whether or not the use of acupuncture combined with conventional treatments improves the prognosis of moderate TH patients compared to conventional treatments alone.

## Methods/Design

### Aims

We hypothesize that the addition of acupuncture to conventional treatments will improve neurological prognosis in TH patients. To test this hypothesis, we will compare study and control groups created based on the size and side of the TH. The study groups will receive acupuncture in addition to standard treatments, while the control groups will receive standard treatments alone.

### Study design

**Fig. 1:**
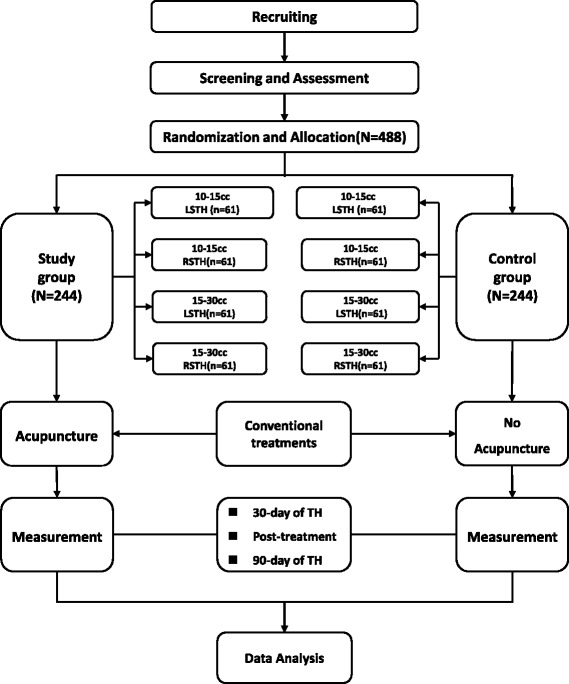
The flow chart. Patients in acupuncture group will receive 36 sessions acupuncture treatment, 6 days a weeks for 6 weeks, in addition to conventional treatments and patients in control group will receive conventional TH treatments only. LSTH, left-sided thalamic hemorrhage; RSTH, right-sided thalamic hemorrhage

This study will be a single-center, randomized, placebo-controlled, parallel group, and patient-assessor-blinded clinical trial. After completing informed consent, 488 eligible patients will be recruited and randomized to one of eight groups: 10–15 cc left sided TH study group (*N* = 61) and a corresponding control group (*N* = 61), 10–15 cc right sided TH study group (*N* = 61) and a corresponding control group, 15–30 cc left sided TH study group (*N* = 61) and a corresponding control group (*N* = 61), and 15–30 cc right sided TH study group (*N* = 61) and a corresponding control group. The patients in each of the study groups will receive 36 sessions of Chinese acupuncture in addition to standard treatments for TH, which include anticonvulsant therapy, antihypertensives, osmotic diuretics for the management of intracranial pressure, and invasive treatments as necessary. The patients in each of the control groups will receive standard treatments only. Patients’ neurologic status will be assessed at baseline and at the end of treatment as well as at 30 day and 90 day follow-up intervals. CT imaging will be performed on admission and at 90 days follow up. All patients will be required to complete written informed consent prior to enrollment. If patients are unable to complete written informed consent, it will be obtained from their relatives. A flow chart demonstrating an outline of the study is included in Fig. 1.

### Study setting

This study will be conducted on a neurosurgery unit in Sichuan University’s West China (Hua Xi) Hospital in Chengdu, China. With 4300 beds and more than 300 surgical operations per day, the hospital is one of the largest in China. The neurosurgery department is the largest in southwest China, with 181 surgical beds, 100 beds for rehabilitation and/or postoperative recovery, and more than 3800 craniotomies performed per year.

### Ethics

This trial will be carried out in accordance with the ethical standards described in the 2013 updated Declaration of Helsinki. The study was approved by the West China Hospital, Sichuan University clinical research and biomedical ethics committees (ethics reference: 2016-044). Written and signed informed consent will be obtained from all participants prior to their inclusion in the study. This trial has also been registered on www.chictr.org.cn (ChiCTR-IOR-16008362) and will be reported in compliance with the CONSORT statement (www.consort-statement.org) [31] as well as STRICTA (Standards for Reporting Interventions in Clinical Trials of Acupuncture) [32].

### Participants

Patients with moderate TH will be recruited from the neurosurgery unit at Huaxi Hospital in China. The diagnosis of TH and the side of the hemorrhage will be determined based on computer tomography. TH volume will be calculated by the neurosurgeon for all patients. Patients who meet eligibility criteria will be enrolled in the study, and written informed consent will be obtained prior to their enrollment.

### Subject enrollment

The nurses responsible for the neurosurgery patients at Huaxi Hospital will be thoroughly educated regarding moderate TH. Patients and/or their legal guardians will be able to contact the project leader at any time should they desire additional information. After obtaining signed informed consent, the project leader will collect certain baseline information (including age, diagnosis, side of hemorrhage (left or right), hematoma volume, medical history, CT report, etc..) and determine whether or not the patient meets the eligibility criteria for the trial or not. Only participants who meet the inclusion criteria and do not possess any of the exclusion criteria will be enrolled. A total of 488 patients with TH who are admitted to the neurosurgical unit of our hospital will be enrolled in the trial.

### Randomization and blinding

Randomization of subjects to the study and control groups will be computer-generated, using the Package for Encyclopaedia Medical Statistics 3.1 (PEMS 3.1) software. Stratified block randomization will not be performed. Concealed allocation will be achieved by having an assigned researcher not to have any contact with patients. Group assignments will be placed in opaque sealed envelopes. Subjects with 10–15 cc left TH, 10–15 cc right TH, 15–30 cc left TH, and 15–30 cc right TH will thus be assigned to either an acupuncture plus conventional treatment group or a conventional treatment alone group, in a 1:1 ratio based on the size and side of their hemorrhage. The primary researchers will be blinded to the type of intervention that each patient has received. The acupuncture practitioners will not provide any clues regarding group assignments to either the researchers or to the statistician.

### Inclusion criteria

Patients with ICH who meet the following inclusion criteria will be enrolled in the trial:

1. First ever TH verified by computer tomography (CT)
2. Moderate TH (10 ~ 30 ml);
3. Unilateral primary TH;
4. Hematoma originating and confined to the thalamus;
5. Age between 18 and 70 years;
6. Acupuncture treatment can be applied within 72 h after TH;
7. Voluntarily willing to sign informed consent.
8. Right handed.
9. All bleeding has stopped at the time of acupuncture initiation.

### Exclusion criteria

Patients meeting any of the following criteria will be excluded from the study:

1. Traumatic TH;
2. Bilateral TH;
3. Hematoma originates in another location and extends to the thalamus or hematoma originates in the thalamus and extends beyond the thalamus;
4. Other areas of cerebral infarction, cerebral hemorrhage, or tumor on admission CT/MRI;
5. Unable to determine the location of the origin of the hematoma on admission CT;
6. Minor TH (<10 ml) or major TH (>30 ml);
7. Heart, liver, or renal failure;
8. Refusal to sign written informed consent;
9. Unable to undergo randomized enrollment.
10. Left handed.
11. Continued bleeding at the time of acupuncture administration.

### Procedure

Eligible patients will be randomized to either study groups (acupuncture plus conventional treatment) or control groups (conventional treatment alone) based on the size and side of their TH. After successful randomization, neurological assessments will occur at four time points: before treatment sessions begin, 30 days after onset of TH, at the end of treatment (6 weeks), and 90 days after onset of TH.

### Intervention

Participants in both groups will receive conventional Western medical treatments as recommended by the guidelines for the management of spontaneous intracerebral hemorrhage including blood pressure management, ICP monitoring, appropriate prophylaxis, and surgical treatment as necessary for life-threatening hemorrhages [2]. In addition to conventional treatment, patients in the study group will receive 36 sessions (once a day, 6 days a week for 6 weeks) of acupuncture.

### Acupuncture

Acupuncture treatment will be started within 72 h after TH onset, and applied once a day, 6 days a week for 6 weeks (a total of 36 sessions). The sessions will be performed by an experienced acupuncture doctor with over 10 years of working experience and 7 years of acupuncture training. This doctor will also receive training prior to the start of the trial. The acupuncture protocol was designed by a Sichuan University Huaxi Hospital professor with more than 25 years of practical experience, in accordance with guidelines from published papers and clinical experience. It will follow the Standards for Reporting Interventions in Clinical Trials for Acupuncture 2010 checklist, as shown in Table 1. Acupuncture points are identified by the point location method as described by the World Health Organization (WHO standard) [33].

**Table 1:** Acupoints to be used in the study

Acupoint	Location	Major indication and function
MS 5 (Dingzhonxian)	At the top of the head, along the middle line of the head, connecting DU 20 to DU 21.	Prolapse, sacral and lumbar problems, paralysis, cortical polyuria, gastroptosis, hyperostosis, hypertension
MS 6 (Dingnieqianxiexian)	1 cun anterior from DU 20 to GB 6.	Treats motor function disorders
MS 7 (Dingniehouxiexian)	1 cun posterior to MS6, from DU 20 to GB 7.	Treats sensory function disorders
LI15 (Jian Yu)	On the shoulder girdle, in the depression between the anterior end of the lateral border of the acromion and the greater tubercle of the humerus.	Shoulder pain, paralysis
LI11 (Qu Chi)	On the lateral aspect of the elbow, at the midpoint of the line connecting LU5 with the lateral epicondyle of the humerus.	Upper extremity palsies, relaxes and strengthens tendons
SJ5 (Wai Guan)	On the posterior aspect of the forearm, at the midpoint of the interosseous space between the radius and the ulna, 2 B-cun proximal to the dorsal wrist crease.	Tinnitus, paralysis of the arms, upper extremity pain;
LI4 (He Gu)	On the dorsum of the hand, in the depression radial and proximal to the second metacarpophalangeal joint.	Spasm in the fingers
ST34 (Liang Qiu)	On the anterolateral aspect of the thigh, between the vastus lateralis muscle and the lateral border of the rectus femoris tendon, 2 B-cun superior to the base of the patella.	Gastrospasm, leg muscle atrophy
ST36 (Zu San Li)	On the anterior aspect of the leg, on the line connecting ST35 with ST41, 3 B-cun inferior to ST35.	Constipation or diarrhea, paralysis of the lower limbs
GB34 (Yang Ling Quan)	On the fibular aspect of the leg, in the depression anterior and distal to the head of the fibula.	Inhibited ability to flex and stretch
SP6 (San Yin Jiao)	On the tibial aspect of the leg, posterior to the medial border of the tibia, 3 B-cun superior to the prominence of the medial Malleolus.	Irregular menstruation, paralysis of the lower limbs
LV3 (Tai Chong)	On the dorsum of the foot, between the first and second metatarsal bones, in the depression distal to the junction of the bases of the two bones, over the dorsalis pedis artery.	Hypertension, paralysis

Both scalp and body acupuncture will be performed in this trial. The parameters for scalp acupuncture will be as follows: two or three needles penetrating through the top midline, the motor region of the affected side, and the sensory region on the side of the lesion. The acupoints on the affected side of the body only and not on the contralateral side will be as follows: LI15 (Jian Yu), LI11 (Qu Chi), SJ5 (Wai Guan), and LI4 (He Gu) in the upper extremity; ST34 (Liang Qiu), ST36 (Zu San Li), GB34 (Yang Ling Quan), SP6 (San Yin Jiao), and LV3 (Tai Chong) in the lower extremity (Table 1). Additional acupoints will be added as follows: RN23 (Lian Quan), bilateral GB20 (Feng Chi), and ST9 (Ren Ying) for dysphagia; ST6 (Jia Che) and ST5 (Di Cang) on the affected side for facial paralysis (Table 2).

**Table 2:** Interventions details according to Standards for Reporting Interventions in Clinical Trials of Acupuncture (STRICTA) guidelines

Item	Detail	Detail response
1. Acupuncture rationale (Explanations and examples)	1a) Style of acupuncture (e.g. Traditional Chinese Medicine, Japanese, Korean, Western medical, Five Element, ear acupuncture, etc)	Traditional needle acupuncture
	1b) Reasoning for treatment provided, based on historical context, literature sources, and/or consensus methods, with references where appropriate	Selected traditional acupuncture points in the 12 meridian system and scalp points based on literature review and clinical experience
	1c) Extent to which treatment was varied	No variation
2. Details of needling (Explanations and examples)	2a) Number of needle insertions per subject per session (mean and range where relevant)	13–19 insertions per session
	2b) Names (or location if no standard name) of points used (uni/bilateral)	Scalp points: motor region and sensory region, unilateral on the side of the lesion Acupoints on limbs:LI15 (Jian Yu), LI11 (Qu Chi), SJ5 (Wai Guan), LI4 (He Gu), ST34 (Liang Qiu), ST36 (Zu San Li), GB34 (Yang Ling Quan), SP6 (San Yin Jiao), LV3 (Tai Chong),all unilateral on the affected side Modification: for dysphagia, GB20 (Feng Chi), RN23 (Lian Quan), and ST9 (Ren Ying); for facial paralysis, ST6 (Jia Che) and ST5 (Di Cang), unilateral on the affected side
	2c) Depth of insertion, based on a specified unit of measurement, or on a particular tissue level	Depth of needle insertion is at least 5 to 10 mm
	2d) Response sought (e.g. *de qi* or muscle twitch response)	De-qi sensation felt by practitioner and subject
	2e) Needle stimulation (e.g. manual, electrical)	Manual
	2f) Needle retention time	30 min
	2 g) Needle type (diameter, length, and manufacturer or material)	Needles: 0.25 × 40 mm, stainless steel (Huatuo brand, made by Suzhou Medical Appliances, China)
3. Treatment regimen (Explanations and examples)	3a) Number of treatment sessions	36 sessions
	3b) Frequency and duration of treatment sessions	6 times per week, interval of 1 day between sessions
4. Other components of treatment (Explanations and examples)	4a) Details of other interventions administered to the acupuncture group (e.g. moxibustion, cupping, herbs, exercises, lifestyle advice)	None
	4b) Setting and context of treatment, including instructions to practitioners, and information and explanations to patients	The same practitioner will treat every subject every session at the patient’s bedside
5. Practitioner background (Explanations and examples)	5) Description of participating acupuncturists (qualification or professional affiliation, years in acupuncture practice, other relevant experience)	Licensed Traditional Chinese medicine doctor at Huaxi Hospital of Sichuan University with more than 10 years of acupuncture treatment experience
6. Control or comparator interventions (Explanations and examples)	6a) Rationale for the control or comparator in the context of the research question, with sources that justify this choice	No acupuncture will be performed on the control group
	6b) Precise description of the control or comparator. If sham acupuncture or any other type of acupuncture-like control is used, provide details as for Items 1 to 3 above.	No sham acupuncture will be performed on the control group

Stimulation will be applied to both the scalp and body acupoints until the patient experiences de qi (obtains qi). A patient’s experience of de qi may take on multiple unique manifestations at the needle site itself and/or around the site of needle manipulation including soreness, aching, numbness, tingling, and even warmth.

Huatuo brand needles (size 0.25 mm × 40 mm) made by Suzhou Medical Appliances in Suzhou, Jiangsu province, China, will be used to perform the acupuncture treatments. Electrical stimulation of acupuncture needles will be done at low frequency (1–3 Hz) in order to ensure patient comfort.

### Control group

Subjects in the control groups will not receive acupuncture treatments. They will receive conventional TH treatments only. Control group participants will then be assessed neurologically at each follow-up visit.

### Dropout criteria

Participants who meet any of the following criteria will be removed from the study: (1) study group subjects who miss more than three sessions (out of a total of 36) of acupuncture; (2) patients who withdraw their consent; (3) patients who experience severe adverse events making their further inclusion in the trial unsustainable; (4) patients whose neurological condition deteriorates making it difficult for them to continue participating in the trial; (5) patients who experience decompensation related to accompanying and/or additional diseases; or (6) patients whose further participation in the trial is felt to be impossible as determined by the principal investigator.

### Outcome measures

#### Primary outcome measure

The primary outcome measure will be NIHSS score, a measure of neurological status, at the end of treatment, at 30 days after TH, and at 90 days after TH. NIHSS is used to describe neurological deficits in stroke patients, and it strongly predicts the likelihood of a patient’s recovery after stroke. The NIHSS is comprised of 11 test items including level of consciousness, gaze, visual field defects, facial palsy, upper extremity motor, lower extremity motor, limb ataxia, sensory ability, language, dysphagia, and neglect. Total score ranges from 0 to 42, with scores above 25 indicating very severe neurological impairment, scores of 5 to 24 indicating moderately severe to severe impairment, and scores below 5 indicating mild impairment.

#### Secondary outcome measures

Secondary outcome measures are as follows:

1. Poor outcome, defined as death or major disability, is defined as a score of 3 to 6 on the modified Rankin scale at 90 days after onset of TH. Scores on the modified Rankin scale range from 0 to 6, with a score of zero indicating no symptoms; a score of five indicating severe disability, confinement to bed, or incontinence; and a score of six indicating death.
2. The rate of surgery at 30 days is defined as the incidence of neurological deterioration requiring surgical intervention.
3. The Glasgow Outcome Scale (GOS) at 90 days after onset of TH is a measure of long-term outcome and includes five grades: 5, good recovery; 4, moderate disability; 3, severe disability; 2, persistent vegetative state; and 1, death.
4. The Barthel activities of daily living (ADL) index and the Rivermead Mobility Index will be performed on each patient at the end of treatment, at 30 days after TH, and at 90 days after TH.
5. Patients with left thalamic hemorrhages will also be evaluated with several language tests including the Boston Naming Test, the Boston Diagnostic Aphasia Exam, the Western Aphasia Battery, and the Mount Wilga High Level Language Screening Test at the end of treatment, at 30 days after TH, and at 90 days after TH.
6. Patients with right thalamic hemorrhages will also be evaluated for visual neglect with the Behavioral In-attention Test (BIT) at the end of treatment, at 30 days after TH, and at 90 days after TH.

### Safety evaluation

Any adverse events or abnormalities will be recorded on case report forms no matter what intervention is used. The severity of such adverse events will be described as mild, moderate, or severe, and the relation of the events to the intervention will be evaluated as not related, possibly related, or related. If any serious adverse events occur as the result of a certain intervention, that intervention will be stopped immediately and appropriate corrective action will be taken. Any serious adverse events will be reported promptly to the institutional review board, according to the protocol.

### Sample size calculation

Using the variable NIHSS score, the primary outcome measure of the study, PS Power and Sample Size software (version 3.0) was used to determine an appropriate sample size for the study. In a previous study, NIHSS scores in each subject group exhibited a normal distribution with a standard deviation of 4.2 points [11]. This assumes a difference of 2.3 points between the groups, as shown in a previous study on moderate TH. A sample size of 488 provides 80% power (α = 0.05) to detect a beneficial effect of early acupuncture therapy on the primary outcome, assuming a 10% non-adherence to treatment and a 3% loss to follow-up.

### Statistical analysis

The results will be presented as mean score plus or minus standard deviation and as percentages. Categorical data will be analyzed with Fisher’s exact test or the Mann-Whitney U test where appropriate. Continuous data will be analyzed with the Student’s t test or Pearson’s test where appropriate. Multi-ranked data will be analyzed using the Mann-Whitney U test. Differences will be considered statistically significant at *P* < 0.05 (two-tailed). All statistical analyses will be performed using the Statistical Package for the Social Sciences (version 13.0; SPSS; Chicago, Illinois, USA).

## Discussion

In this randomized controlled trial, we will observe the effect of acupuncture therapy on patients with acute TH. The findings of this project are expected to provide evidence for the efficacy of acupuncture in improving the prognosis of patients with moderate TH. To minimize bias, stratified block randomization will be used according to sex and age, and the outcomes reviewer will be blinded. Subjects will be treated alone in a treatment room in order to avoid any communication with other subjects.

Generally speaking, no ideal placebo-controlled acupuncture trials have been performed previously [34]. Because recent acupuncture trials have examined so-called sham acupuncture techniques, including: needling of acupuncture points through non-penetrating needles, needling of non-acupuncture points, and needling of acupuncture points that are not indicated for that specific condition, these prior trials have not accurately reflected TCM theory and therefore have not properly assessed it [34]. Further complicating things is that sham acupuncture has been shown to have some efficacy [35]. In China, many patients and their families have had acupuncture at some time in their lives or are at least familiar with the acupuncture process. Therefore, it would be difficult to design a trial assessing the utility of sham acupuncture versus real acupuncture while keeping the patient blinded.

The decision was made to examine patients with left sided TH separately from patients with right sided TH. In the majority of patients, Broca’s cortical area and Wernicke’s cortical area are located in the left hemisphere. Therefore, patients with left hemisphere stroke (ischemic or hemorrhagic) are at risk for language deficits. Similarly, patients with right sided insults are at risk for symptoms of visual neglect. Both language deficits and visual neglect can have a profound impact on the way a patient responds to treatment and rehabilitation of other neurologic deficits. Therefore, to account for the potentially confounding effects of language deficits and visual neglect respectively, patients were subdivided into right and left TH subgroups.

Along the same lines, not all patients have their language centers located in their left hemisphere. While the majority of left handed patients have their language centers in the left hemisphere, some do have them in the right hemisphere. Therefore, in this study, we have only included right handed patients.

Another potentially confounding factor is the involvement of adjacent brain tissue as a secondary result of the initial thalamic insult. Adjacent brain tissue can necrose either directly via extension of the thalamic bleed or indirectly via mass effect from the thalamic bleed and/or edema related to the thalamic bleed. Areas at specifically high risk include the genu of the internal capsule which lies lateral to the thalamus, the posterior limb of the internal capsule which lies posterolateral to the thalamus, the anterior limb of the internal capsule which lies anterolateral to the thalamus, and the peri-thalamic/peri-ventricular white matter which lies superior to the thalamus. Involvement of the genu of the internal capsule, the posterior limb of the internal capsule, and adjacent white matter could result in paralysis. Involvement of the posterior limb of the internal capsule could also result in sensory loss or impaired comprehension [36]. Finally, involvement of the anterior limb of the internal capsule could result in cognitive issues. Involvement of any of these areas could potentially impact how a patient recovers and/or sway the post-stroke assessment scores in one direction or another depending on how many of the affected patients ended up in the control group or the intervention group. To combat this, we utilized two mechanisms. First, we excluded patients from the study whose TH on admission CT demonstrated extension of the insult beyond the thalamus. However, admission CT often does not disclose the true area affected by the insult. CT performed 90 days later is much more sensitive in demonstrating the insult’s true encompassment. We therefore further subdivided the groups by size. While all subjects in the study have moderate thalamic hemorrhage, groups were further subdivided into subjects with 10–15 cc of TH and 15–30 cc of TH. Patients with 10–15 cc of TH are much less likely to experience insults to brain tissue external to the thalamus than patients with 15–30 cc of TH as a result of the smaller size of the initial bleed. We also plan to retrospectively analyze the results of CT scans performed 90 days following the insult to determine what percent of patients in each group experienced extension of their primary insult to tissue beyond the thalamus and how this may have affected the results in that group. After dividing patients by side of hemorrhage and size of hemorrhage four intervention groups were established, and along with the corresponding four control groups, this totaled eight distinct subject groups.

Previous research has demonstrated that patients with hand paresis only following stroke may not only be the most likely to benefit from acupuncture therapy but also the most likely to experience complete resolution of their neurologic symptoms [37]. As a result, it is important, if able, to determine the results of our intervention in patients with hand paresis only. As only patients with moderate thalamic hemorrhage will be enrolled in this trial, it is unlikely that our study will include many, if any, subjects with hand paresis only. However, if there are subjects with hand paresis, the results of our intervention will be analyzed retrospectively following conclusion of the trial.

The primary outcome measure is NIHSS result. While the NIHSS is a fine test, its comprehensiveness is somewhat lacking. In fact, previous studies examining interventions in stroke patients may have failed due to their sole reliance on the NIHSS as an outcome measure [38]. Therefore, additional tests measuring paralysis, language, visual neglect, and stroke outcome were also included in this study.

Under strict quality control, this trial will attempt to answer the question of whether or not acupuncture can improve neurologic outcome following moderate TH.

## Trial status

Recruitment commenced in January 2017, and it is anticipated that the trial will be completed by March 2021.

## Abbreviations

CT: Computer tomography
GOS: Glasgow outcome scale
ICH: Intracranial hemorrhage
ICP: Intracranial pressure
MRI: Magnetic resonance imaging
STRICTA: Standards for Reporting Interventions in Clinical Trials of Acupuncture
TCM: Traditional Chinese Medicine
TH: Thalamic hemorrhage
WHO: World Health Organization

## References

[1] Lee SH, Park KJ, Kang SH, Jung YG, Park JY, Park DH (2015). Prognostic Factors of Clinical Outcomes in Patients with Spontaneous Thalamic Hemorrhage. Med Sci Monit.

[2] Hemphill JC, Greenberg SM, Anderson CS, Becker K, Bendok BR, Cushman M, Fung GL, Goldstein JN, Macdonald RL, Mitchell PH (2015). Guidelines for the Management of Spontaneous Intracerebral Hemorrhage: A Guideline for Healthcare Professionals From the American Heart Association/American Stroke Association. Stroke.

[3] Broderick J, Connolly S, Feldmann E, Hanley D, Kase C, Krieger D, Mayberg M, Morgenstern L, Ogilvy CS, Vespa P (2007). Guidelines for the management of spontaneous intracerebral hemorrhage in adults: 2007 update: a guideline from the American Heart Association/American Stroke Association Stroke Council, High Blood Pressure Research Council, and the Quality of Care and Outcomes in Research Interdisciplinary Working Group. Circulation.

[4] Greenberg M, Arredondo N (2006). Handbook of neurosurgery.

[5] van Asch CJ, Luitse MJ, Rinkel GJ, van der Tweel I, Algra A, Klijn CJ (2010). Incidence, case fatality, and functional outcome of intracerebral haemorrhage over time, according to age, sex, and ethnic origin: a systematic review and meta-analysis. Lancet Neurol.

[6] Gaab MR (2011). Intracerebral hemorrhage (ICH) and intraventricular hemorrhage (IVH): improvement of bad prognosis by minimally invasive neurosurgery. World Neurosurg.

[7] Kanno T, Sano H, Shinomiya Y, Katada K, Nagata J, Hoshino M, Mitsuyama F (1984). Role of surgery in hypertensive intracerebral hematoma: a comparative study of 305 nonsurgical and 154 surgical cases. J Neurosurg.

[8] Nakano T, Ohkuma H (2005). Surgery versus conservative treatment for intracerebral haemorrhage—is there an end to the long controversy?. Lancet.

[9] Cho DY, Chen CC, Lee HC, Lee WY, Lin HL (2008). Glasgow Coma Scale and hematoma volume as criteria for treatment of putaminal and thalamic intracerebral hemorrhage. Surg Neurol.

[10] Mori S, Sadoshima S, Ibayashi S, Fujishima M, Iino K (1995). Impact of thalamic hematoma on six-month mortality and motor and cognitive functional outcome. Stroke.

[11] Chen M, Wang Q, Zhu W, Yin Q, Ma M, Fan X, Li Y, Ni G, Liu C, Liu W (2012). Stereotactic aspiration plus subsequent thrombolysis for moderate thalamic hemorrhage. World Neurosurg.

[12] Keep RF, Hua Y, Xi G (2012). Intracerebral haemorrhage: mechanisms of injury and therapeutic targets. Lancet Neurol.

[13] Xi G, Keep RF, Hoff JT (2006). Mechanisms of brain injury after intracerebral haemorrhage. Lancet Neurol.

[14] Aronowski J, Zhao X (2011). Molecular pathophysiology of cerebral hemorrhage: secondary brain injury. Stroke.

[15] Gebel JM, Jauch EC, Brott TG, Khoury J, Sauerbeck L, Salisbury S, Spilker J, Tomsick TA, Duldner J, Broderick JP (2002). Relative Edema Volume Is a Predictor of Outcome in Patients With Hyperacute Spontaneous Intracerebral Hemorrhage. Stroke.

[16] Qureshi AI, Mendelow AD, Hanley DF (2009). Intracerebral haemorrhage. Lancet.

[17] Mehdiratta M, Kumar S, Hackney D, Schlaug G, Selim M (2008). Association between serum ferritin level and perihematoma edema volume in patients with spontaneous intracerebral hemorrhage. Stroke.

[18] World Health Organization. Acupuncture: review and analysis of reports on controlled clinical trials. Geneva; 2002.

[19] Chen F, Qi Z, Luo Y, Hinchliffe T, Ding G, Xia Y, Ji X (2014). Non-pharmaceutical therapies for stroke: mechanisms and clinical implications. Prog Neurobiol.

[20] He T, Zhu W, Du SQ, Yang JW, Li F, Yang BF, Shi GX, Liu CZ (2015). Neural mechanisms of acupuncture as revealed by fMRI studies. Auton Neurosci.

[21] Chen JC (2015). The effects of acupuncture and traditional Chinese medicines on apoptosis of brain tissue in a rat intracerebral hemorrhage model. Physiol Behav.

[22] Litscher G (2009). Ten years evidence-based high-tech acupuncture—a short review of peripherally measured effects. Evid Based Complement Alternat Med.

[23] Li HQ, Li JH, Liu AJ, Ye MY, Zheng GQ (2014). GV20-based acupuncture for animal models of acute intracerebral haemorrhage: a preclinical systematic review and meta-analysis. Acupunct Med.

[24] Zou W, Chen QX, Sun XW, Chi QB, Kuang HY, Yu XP, Dai XH (2015). Acupuncture inhibits Notch1 and Hes1 protein expression in the basal ganglia of rats with cerebral hemorrhage. Neural Regen Res.

[25] Luo JK, Zhou HJ, Wu J, Tang T, Liang QH (2013). Electroacupuncture at Zusanli (ST36) accelerates intracerebral hemorrhage-induced angiogenesis in rats. Chin J Integr Med.

[26] Cho NH, Lee JD, Cheong BS, Choi DY, Chang HK, Lee TH, Shin MC, Shin MS, Lee J, Kim CJ (2004). Acupuncture suppresses intrastriatal hemorrhage-induced apoptotic neuronal cell death in rats. Neurosci Lett.

[27] Sun F, Wang J, Wen X (2012). Acupuncture in stroke rehabilitation: Literature retrieval based on international databases. Neural Regen Res.

[28] Wu HM, Tang JL, Lin XP, Lau J, Leung PC, Woo J, Li Y. Acupuncture for stroke rehabilitation. Cochrane Database Syst Rev. 2006;3. 10.1002/14651858.CD004131.pub2 16856031

[29] Ernst E, Lee MS (2010). Acupuncture during stroke rehabilitation. Stroke.

[30] Zhuang L, He J, Zhuang X, Lu L (2014). Quality of reporting on randomized controlled trials of acupuncture for stroke rehabilitation. BMC Complement Altern Med.

[31] Schulz KF, Altman DG, Moher D, Group C (2010). CONSORT 2010 Statement: updated guidelines for reporting parallel group randomised trials. BMC Med.

[32] MacPherson H, Altman DG, Hammerschlag R, Youping L, Taixiang W, White A, Moher D (2010). Revised standards for reporting interventions in clinical trials of acupuncture (STRICTA): extending the CONSORT statement. J Evid Based Med.

[33] Pacific WROftW (2008). WHO standard acupuncture point locations in the Western Pacific Region.

[34] Appleyard I, Lundeberg T, Robinson N (2014). Should systematic reviews assess the risk of bias from sham–placebo acupuncture control procedures?. Eur J Integr Med.

[35] Moffet HH (2009). Sham acupuncture may be as efficacious as true acupuncture: a systematic review of clinical trials. J Altern Complement Med.

[36] Naeser MA, Palumbo CL (1994). Neuroimaging and language recovery in stroke. J Clin Neurophysiol.

[37] Naeser MA, Alexander MP, Stiassny-Eder D, Galler V, Hobbs J, Bachman D (1994). Acupuncture in the treatment of paralysis in chronic and acute stroke patients--improvement correlated with specific CT scan lesion sites. Acupunct Electrother Res.

[38] Zivin JA, Sehra R, Shoshoo A, Albers GW, Bornstein NM, Dahlof B, Kasner SE, Howard G, Shuaib A, Streeter J, Richieri SP, Hacke W (2014). NeuroThera efficacy and safety trial-3 (NEST-3). Int J Stroke.

